# Impact of a malaria intervention package in schools on *Plasmodium* infection, anaemia and cognitive function in schoolchildren in Mali: a pragmatic cluster-randomised trial

**DOI:** 10.1136/bmjgh-2016-000182

**Published:** 2017-06-28

**Authors:** Siân E Clarke, Saba Rouhani, Seybou Diarra, Renion Saye, Modibo Bamadio, Rebecca Jones, Diahara Traore, Klenon Traore, Matthew CH Jukes, Josselin Thuilliez, Simon Brooker, Natalie Roschnik, Moussa Sacko

**Affiliations:** 1 Faculty of Infectious and Tropical Diseases, London School of Hygiene & Tropical Medicine, London, UK; 2 Save the Children, Bamako, Mali; 3 Ministry of Health, Institut National de Recherche en Santé Publique, Bamako, Mali; 4 Ministry of Health, Programme National de Lutte contre le Paludisme, Bamako, Mali; 5 RTI International, Research Triangle Park, North Carolina, USA; 6 CNRS (Centre National de la Recherche Scientifique) - Centre d'Économie de la Sorbonne, Paris, France

**Keywords:** malaria, anaemia, cognition, schoolchildren, schools, insecticide-treated nets, ITNs. LLINs, intermittent preventive treatment, IPT, intermittent parasite clearance, IPC, asymptomatic, gametocytes, Mali

## Abstract

**Background:**

School-aged children are rarely targeted by malaria control programmes, yet the prevalence of *Plasmodium* infection in primary school children often exceeds that seen in younger children and could affect haemoglobin concentration and school performance.

**Methods:**

A cluster-randomised trial was carried out in 80 primary schools in southern Mali to evaluate the impact of a school-based malaria intervention package. Intervention schools received two interventions sequentially: (1) teacher-led participatory malaria prevention education, combined with distribution of long-lasting insecticidal nets (LLINs), followed 7 months later at the end of the transmission season by (2) mass delivery of artesunate and sulfadoxine-pyrimethamine administered by teachers, termed intermittent parasite clearance in schools (IPCs). Control schools received LLINs as part of the national universal net distribution programme. The impact of the interventions on malaria and anaemia was evaluated over 20 months using cross-sectional surveys in a random subset of 38 schools(all classes), with a range of cognitive measures (sustained attention, visual search, numeracy, vocabulary and writing) assessed in a longitudinal cohort of children aged 9–12 years in all 80 schools.

**Results:**

Delivery of a single round of IPCs was associated with dramatic reductions in malaria parasitaemia (OR 0.005, 95% CI 0.002 to 0.011, p<0.001) and gametocyte carriage (OR 0.02, 95% CI 0.00 to 0.17, p<0.001) in intervention compared with control schools. This effect was sustained for 6 months until the beginning of the next transmission season. IPCs was also associated with a significant decrease in anaemia (OR 0.56, 95% CI 0.40 to 0.78, p=0.001), and increase in sustained attention (difference +0.23, 95% CI 0.10 to 0.36, p<0.001). There was no evidence of impact on other cognitive measures.

**Conclusion:**

The combination of malaria prevention education, LLINs and IPCs can reduce anaemia and improve sustained attention of school children in areas of highly seasonal transmission. These findings highlight the impact of asymptomatic malaria infection on cognitive performance in schoolchildren and the benefit of IPCs in reducing this burden. Additionally, malaria control in schools can help diminish the infectious reservoir that sustains *Plasmodium* transmission.

**Key questions**

**What is already known about this topic?**

- Asymptomatic infection with *Plasmodium* parasites is common in school-aged children in Africa.
- Asymptomatic infections increase the risk of anaemia and have been associated with reductions in cognitive function.
- While deleterious effects of malaria infection on cognition could translate into lower school performance, this has yet to be demonstrated.

**What are the new findings?**

- This trial found that malaria control delivered through schools can significantly reduce the risk of anaemia and improve sustained attention in primary school children.
- These data provide the first evidence from an area of highly seasonal transmission and corroborate findings from earlier trials of malaria parasite clearance in schools.
- Improvements in health outcomes lasted until the beginning of the next transmission season and indicate that for schoolchildren in the sub-Sahel region, a single annual intermittent parasite clearance treatment given in schools at the end of the transmission season may be sufficient to have a prolonged benefit spanning the entire schoolyear.

**Recommendations for policy**

- Malaria control initiatives in schools have potential to generate multiple benefits—improving health and supporting educational progress—and should be an integral component of education and school health plans in malaria-endemic countries.
- Equally, national malaria control strategy should not overlook this age group.
- School-aged children comprise a sizeable proportion of the population, and by diminishing the infectious reservoir that sustains transmission, malaria control in schools could have an additional role in transmission-reduction strategies.

## Introduction

School-aged children are rarely targeted by malaria control, yet the prevalence of *Plasmodium* infection in this age group often exceeds that seen in younger children.^1–3^ It can also be anticipated that current successes in lowering transmission may, through delaying the acquisition of immunity, lead to an increase in the incidence of clinical malaria in school-aged children in previously highly endemic areas in the future.^4 5^ Malaria among school children has received increasing research attention over the last 10 years, with an expanding body of knowledge on the negative impacts that asymptomatic infection can have on health and education, as well as the deleterious effects of clinical attacks in this group.^6–19^ Nonetheless, there remains a paucity of evidence on the optimal control strategies in schoolchildren, and how these might vary between different malaria transmission settings.^20 21^ Intermittent preventive treatment in schools has previously been shown to reduce malaria infections and anaemia and to improve sustained attention in an area of intense perennial transmission in western Kenya^12^ but has not been evaluated in areas of seasonal transmission. Seasonal malaria chemoprevention in the Sahel region, when expanded to target children up to 10 years of age, is associated with significant improvement in malaria and anaemia in all ages^13 22^ but impacts on cognition or learning have not been examined. We undertook a cluster-randomised trial, in an area of highly seasonal malaria in southern Mali, to investigate the impact of a comprehensive malaria control strategy in schools, which combined vector control using long-lasting insecticidal nets (LLINs) supported by teacher-led participatory malaria prevention education and mass treatment to clear residual parasitaemia (termed intermittent parasite clearance in schools (IPCs)), in reducing malaria infections and anaemia and improving children’s capacity to pay attention in class.

## Methods

### Study area and population

A two-arm stratified cluster-randomised trial was carried out in primary schools in Sikasso region, south-eastern Mali, to inform Save the Children’s school health and nutrition programme that aims to address key health problems that prevent children from participating and learning in school. The region is characterised by high humidity and rainfall, with an estimated entomological inoculation rate (EIR) in excess of 100 infective bites per annum.^23^ Malaria transmission is highly seasonal, and the malaria risk is concentrated within a 6-month period between May and November. Sikasso is the region most affected by malaria: in August–November 2010, 59% of 229 children under 5 years were infected with *Plasmodium falciparum*; 92% were anaemic (<110 g/L); and 35% had severe anaemia (<80 g/L).^24^ No comparable data were available for school-aged children.

### Participants

Sample size calculations for each of the primary biomedical and cognitive outcomes, taking clustering of students within schools into account, were carried out to determine the number of schools and students required.^25^ Assuming a 48% prevalence of anaemia (primary biomedical outcome) at baseline and an intraclass correlation of 0.025, it was estimated that a sample size of 50 students in 19 schools per study arm (a total of 1900 students) would provide 90% power to detect a 25% reduction in anaemia in the intervention arm at the 5% level of statistical significance. For cognitive outcomes, it was estimated that a minimum number of 40 schools in each arm, with 15 children per school, was required to detect an effect size of 0.2 SD in sustained attention, assuming an intraclass correlation of 0.15 and a correlation of 0.7 between values measured at baseline and endline.

Rural government or community primary schools located within the Sikasso administrative region, with a total enrolment of 50 pupils or more, were eligible for inclusion in the study. Any school that lacked both classes 4 and 5 at baseline was not eligible to participate in the trial. A total of 114 schools met the eligibility criteria and were stratified into four groups according to the previous academic year’s pass rate (80%–100%; 60%–80% 0%–60%; no information), to account for differences in school quality and socioeconomic environment. Within each school performance stratum, 20 schools were randomly selected for inclusion in the trial. At this stage, one school refused to participate and was replaced with another school using the same selection method. Schools were randomly allocated to either the control or intervention arm; 40 schools per arm. Selection of schools and allocation to study arm were performed using a random number generator in Microsoft Excel. Cognitive outcomes were assessed at baseline and follow-up in all 80 schools randomised to the trial. Within each study arm, a subsample of 19 schools were randomly selected for inclusion in the evaluation of biomedical outcomes at baseline and follow-up (total=38 schools, 19 per arm).

### Intervention

All children enrolled in the 40 intervention schools received two malaria interventions sequentially: (1) teacher-led participatory malaria prevention education in schools, combined with school-based distribution of LLINs, prior to the start of the malaria transmission season, followed by (2) intermittent parasite clearance in schools (IPCs) at the end of the transmission season. Control schools received LLINs as part of the national universal net distribution programme.

#### Malaria prevention education and net distribution

All teachers were trained on malaria-specific knowledge, demonstrations of bednet use and participatory classroom activities based on the Child-to-Child approach to help incorporate malaria education into schooling during a 3-day workshop. Each participant received a teacher’s manual (available on request from the authors). Key messages in the education programme included the possible adverse effects of malaria in school age children, role of mosquitoes in transmitting malaria, advantages of insecticide treated nets and importance of continued net use throughout the year. In April 2011, teachers began the participatory malaria education activities in schools and organised a public event at which two LLINs were issued to every child in the 40 intervention schools, for use by the target child, as well as other household members.

#### Intermittent parasite clearance in schools (IPCs)

In December 2011, children in intervention schools also received intermittent parasite clearance, in which a treatment dose was given to all pupils irrespective of infection status, timed to coincide with the end of the malaria transmission season. IPCs comprised a single annual treatment of artesunate plus sulfadoxine-pyrimethamine (AS/SP), which was administered in school by teachers over three consecutive days. Dosage was given according to age: 5–6 years: 1 tablet AS (50 mg) daily for 3 days+1 tablet SP (500/25 mg) on day 1 only; 7–13 years: two tablets AS (50 mg) daily+2 tablets SP (500/25 mg) on day 1. Exclusion criteria included self-reported pregnancy, prior history of adverse reaction to sulfa-based drugs or current treatment for symptomatic malaria. One-day training on IPCs drug administration and monitoring was provided for all teachers. Children were observed and treatment was readministered if the child vomited or spat out the tablets within 30 min of drug administration. Pupils who were absent on the first day of treatment were excluded from treatment. Pupils who started treatment but were absent on subsequent days were traced and treated at home; if they could not be found at home, they were excluded from the remainder of the treatment. Adverse events were monitored by teachers for 7 days after treatment and referred to the local health facility.

In May 2011, the National Malaria Control Program in Mali implemented a community-based LLIN distribution programme throughout Sikasso region, which aimed to achieve universal coverage by providing one net for every two people. The universal campaign was planned and implemented after the beginning of the school-based trial and covered all intervention and control villages enrolled in the trial.

### Evaluation of impact

As both study arms received long-lasting insecticidal nets via the government programme, study outcomes were of necessity compared between schoolchildren in schools receiving the comprehensive school-based malaria control programme (LLINs, education and IPCs) and schoolchildren in communities receiving LLINs through the universal coverage campaign alone. The primary biomedical endpoint was prevalence of anaemia. The primary cognitive endpoint was mean score in tests of sustained attention, chosen as less dependent on prior learning than other tasks in the cognitive battery. Secondary endpoints included prevalence and intensity of *Plasmodium* infection, mean haemoglobin concentration and performance in a range of cognitive tests. Cross-sectional surveys were carried out at baseline in November 2010 and repeated at two subsequent time points. Data gathered in November 2011 after the first phase of the intervention were used to assess the impact of the school bednet distribution and malaria education programme, while outcomes collected in February 2012 after the second phase of the intervention were used to evaluate the combined impact of the IPCs treatment, bednet distribution and malaria education programme. To preserve blinding, staff responsible for measuring study outcomes were unaware of group allocation, and data analysis was undertaken by a statistician with no prior involvement in the trial.

### Cognitive outcomes

The effect of the intervention on cognitive abilities was investigated by comparing differences in test scores given to a cohort of students in classes 4 and 5 (typically aged 10–12 years). This age group was selected for testing because they were old enough to understand the test instructions and could thus be assessed in groups. In each of the 80 schools, 15 students were randomly selected from class 4 at baseline. In schools without a class 4, 15 children were selected from class 5. The sample was drawn using a number counting system on the day of the survey: to ensure gender balance pupils were separated by sex, and the number of boys (or girls) divided by 15 to yield a ‘selection number’. Children of each sex were lined up in a random order and counted off with each child on whom the ‘selection number’ landed chosen for participation. The same children were followed longitudinally over the course of the study.

Sustained attention was evaluated using two code transmission tasks, adapted from the TEA-Ch (Test of Everyday Attention for Children) battery^26^ for group administration in groups of 15 or fewer, as used in a previous trial in Kenya.^12^ Both tests involved listening to a prerecorded list of digits read aloud at the speed of one per second. Children were required to listen out a ‘code’—two consecutive occurrences of the number 5—and to write down the number (single-digit test) or two numbers (double-digit test) which immediately preceded the code. Secondary cognitive outcomes were examined using a set of tasks previously used in Mali to assess understanding of word meaning (vocabulary test in French), writing and numeracy skills and a visual search task.^14^ For these outcomes, two versions of each task were used: with an easier and/or shorter version administered to pupils in class 4, and a more difficult or longer version administered to pupils in class 5.

### Biomedical outcomes

**Figure 1:**
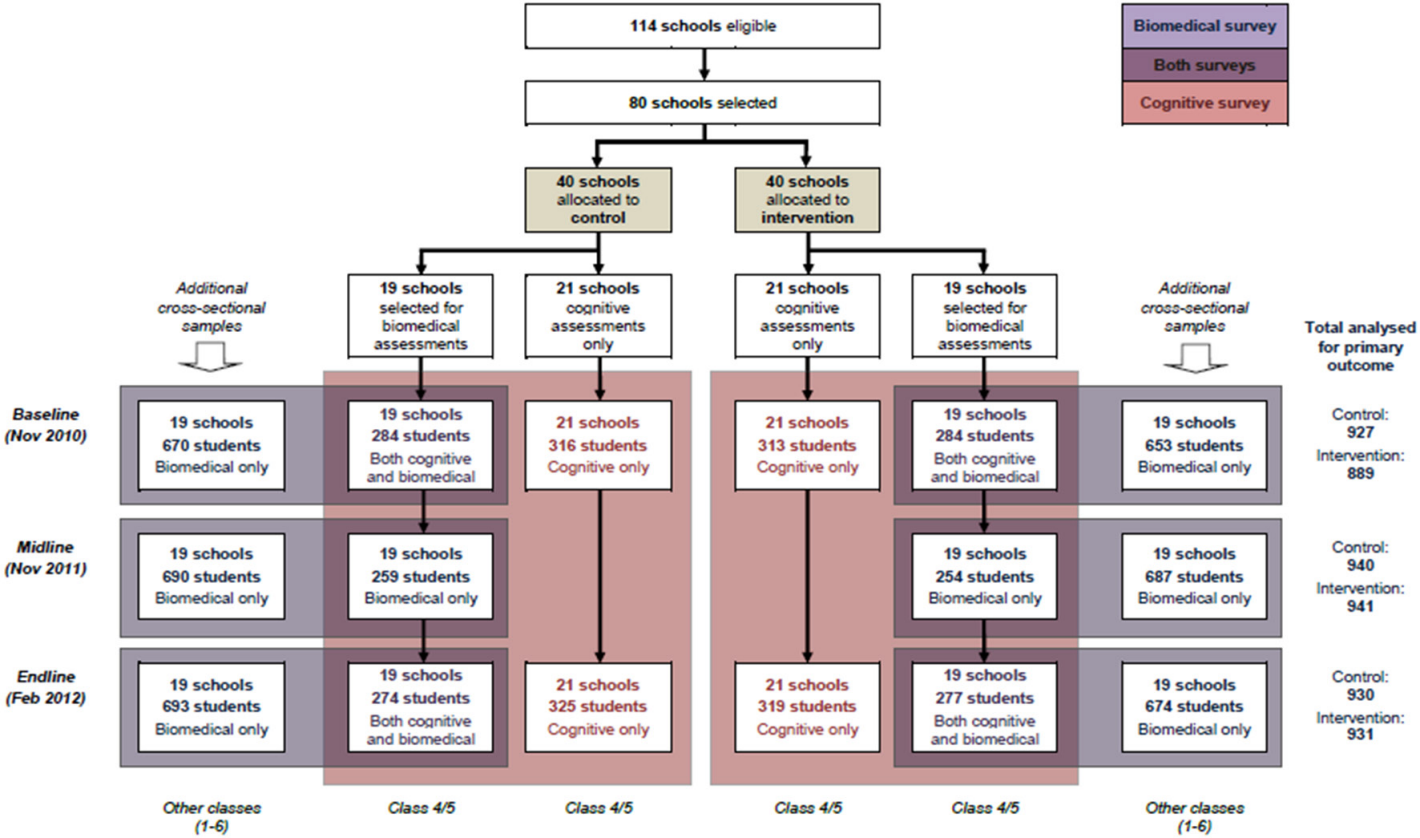
Trial profile showing number of schoolchildren surveyed at each time point, by study arm.

Biomedical outcomes were assessed, on the day following the cognitive assessments, in a randomly selected subset of 38 schools. The biomedical sample included the full age range of primary school pupils (age 5–18 years) and comprised the 15 pupils already enrolled in the cognitive cohort, plus an additional 35 pupils randomly selected from among the rest of the school (excluding the class from which the longitudinal sample had been drawn). The sample was drawn on the day of the survey using a number counting system, as described above, with the total number of boys (or girls) divided by 35 to yield a ‘selection number’. This sampling procedure was undertaken at baseline and repeated afresh in November 2011 and February 2012. In this manner, data were collected at each time point on approximately 1900 students (50 per school, 950 in each study arm). As a result of the selection procedure, the biomedical dataset available for analysis included some data collected from the same 570 children evaluated at all three time points (15 per school in cognitive cohort) and a larger majority of data collected from mainly different individuals at each time point (1330 children; 35 per school). The data available from each time point are summarised in figure 1.

Finger-prick blood samples were taken to assess haemoglobin concentration (Hb) with a portable photometer (Hemocue, Angelholm, Sweden). Anaemia was defined according to WHO age-specific thresholds: Hb <110 g/L for children aged less than 5 years, Hb <115 g/L for children aged 5–11 years, Hb <120 g/L for children aged 12–14 years and female children >15 years and Hb <130 g/L for boys aged >15 years.^27^ Thick and thin blood smears were prepared for malaria parasites, stained for 15 min with 10% Giemsa and examined by light microscopy by an experienced research technician. Slides were declared negative after examination of 100 negative high-powered fields. Microscopy was repeated on a 10% random sample of slides for quality assurance. Height (cm) and weight (kg) were measured and anthropometric indices height-for-age, weight-for-age, weight-for-height, and body mass index, calculated using the WHO AnthroPlus software.^28^ Data on malaria knowledge and bed net use, as well as covariates such as household socioeconomic indicators and parental literacy, were obtained through a questionnaire administered to students in classes 3–6 at the time of the biomedical surveys.

### Cross-sectional survey in May 2012

Following the final evaluation in February 2012, intermittent parasite clearance was carried out in control schools. To investigate how long the effects of the intervention might persist, an additional cross-sectional biomedical survey was undertaken at the end of the school year in May 2012 in a randomly selected subsample of five schools in the intervention arm (250 schoolchildren). Malaria parasitaemia and anaemia were compared with 250 children in five randomly selected schools that had not previously participated in the study.

### Statistical analysis

For each biomedical outcome (anaemia, mean Hb, *Plasmodium* infection), separate statistical models were fitted to assess (1) the impact of the school bed nets and malaria education programme (using data from the first follow-up survey in November 2011) and (2) the combined impact of the IPCs treatment, bed nets and malaria education programme (using data from the second follow-up survey in February 2012). For the cognitive outcomes (sustained attention, vocabulary, writing, numeracy and visual search skills), only the full intervention including the IPCs treatment was evaluated.

For each outcome, data from baseline and the relevant follow-up were analysed in a single statistical model irrespective of whether students had provided data at both or just one of these time points. This enabled data from longitudinal and repeat cross-sectional samples to be analysed together and is analogous to adjusting for baseline using analysis of covariance. The analysis was performed using mixed effects models with a random effect of school to account for correlations among students in the same school and a random effect of student to account for repeated measures over time within the longitudinal cohort. Fixed effects were study arm (intervention vs control), time (follow-up vs baseline) and the interaction between study arm and time. The key effect of interest is the interaction, which estimates the change from baseline to follow-up in the intervention arm relative to change in the control arm. All analyses accounted for the nature of the distribution of the outcome and appropriate summary statistics and measures of effect are reported.

For cognitive outcomes, test scores were standardised prior to analysis to create a z-score using the mean and SD of each measure at baseline. Potential practice effects were controlled for by including in analyses a covariate which indicated whether the student had already experienced the test on a prior occasion. For those outcomes where versions of the task differed between class 4 and 5, z-scores were calculated separately by class, and analysis controlled for variations in difficulty and scoring between different versions of the test used, and interactions between version and both practice and time. Where test scores were not normally distributed and demonstrated floor or ceiling effects, bias corrected bootstrap 95% CI estimated from 2000 bootstrap replications are reported, and statistical significance at p=0.05 is inferred from the 95% CI.

For the biomedical outcomes, an adjusted analysis, which controlled additionally for age, sex, height for age z-score and village level prevalence of malaria parasitaemia at baseline (proxy for local risk of infection), was carried out. Since this full set of covariates was not collected from every student in the larger cognitive sample (80 schools), adjusted analyses for cognitive outcomes controlled only for age, sex and school class.

A supplementary analysis on the smaller subset of students for whom both biomedical and cognitive measures were available (38 schools) was also carried out. The adjusted analysis for this subset controlled for parental literacy, socioeconomic status (as measured by ownership of household assets), height for age z-score and village level prevalence of malaria parasitaemia at baseline, in addition to age, sex and school class.

All analysis was by intention to treat and was carried out using Stata V.13.1.

### Ethical considerations

Ethical approval for the trial was granted by the Ethical Committee of the National Institute of Research in Public Health in Mali (00013/10/CE-INRSP). Community consent was obtained at the time of enrolment into the trial. Sensitisation meetings were held with village leaders, school staff and members of the school management committees (Comité de Gestion Scolaire) and read a standardised script detailing the justification, procedures, risks and benefits of the trial. Parents and students were informed of their right to withdraw from the study at any time. In communities agreeing to participate in the trial, written consent was provided by the president of the school management committee. Prior to each of the two follow-up surveys, community meetings were repeated to brief parents about the surveys, and verbal assent was sought from each student before participation.

Students presenting with clinical malaria on the day of the surveys, defined as fever and a positive rapid diagnostic test, were started on artemisinin combination therapy with Coartem. Parents, older siblings or teachers were briefed to ensure the completion of the 3-day regimen. Students with severe anaemia, defined as a haemoglobin concentration of less than 70 g/L, were immediately given iron supplements and referred to the nearest health facility. Students with severe malaria or symptoms of other serious ailments were also referred.

The trial was registered with current controlled trials (ISRCTN26838440; Pre-results; http://www.controlled-trials.com/ISRCTN26838440).

### Role of the funding source

The trial was conducted by Save the Children in partnership with the Ministry of Health and Ministry of Education, with funding from Save the Children raised through child sponsorship. Analysis was undertaken by an independent statistician at LSHTM with no prior involvement in the trial, according to a predefined analytical plan. SEC, SR, RJ, JT, MS, NR and SB had full access to the data and made the final decision to submit for publication.

## Results

### Trial profile and baseline data

Prior to the intervention, only 41.7% of schoolchildren slept under a bednet: 79.8% were infected with malaria parasites and 62.9% were anaemic at the time of the biomedical cross-sectional survey in November 2010, with malaria prevalence in schools ranging between 52% and 94%. Overall, the two study arms were well balanced at baseline (table 1a and b). For three schools, many of the blood slides were inadvertently destroyed (two controls and one intervention), resulting in an imbalance in the amount of missing data for *Plasmodium* infection (8.9% and 4.1% missing in the control and intervention groups, respectively) and parasite density (9.0% and 5.2% missing). For all other measures, the quantity of missing data was low (<6%) and similar in both study arms. The prevalence of *Plasmodium* infection at baseline was similar in the two arms, although parasite density was marginally higher among infected children in the control schools. Reported household ownership of mopeds and telephones was slightly higher in the intervention arm. Nonetheless, biomedical outcomes at baseline were generally similar in the two study arms (table 1a). Among children enrolled into the cognitive cohort, the mean age of pupils in the intervention arm was slightly higher, with a higher proportion of the sample being in class 5; however, test scores in the battery of tests at baseline were similar (table 1b). For all measures, the quantity of missing data was low (<1%) in both arms of the cognitive cohort. In this older group of children, the prevalence of anaemia was slightly lower (57%), though malaria parasitaemia remained in excess of 74%; all biomedical parameters at baseline were similar in both arms (see online supplementary table S1).

10.1136/bmjgh-2016-000182.supp1 Supplementary Table 1

**Table 1a:** Characteristics of children in control and intervention schools at baseline: November 2010 (classes 1–6)

	Control schools	Intervention schools
Number of schools surveyed	19	19
Number of children surveyed	954	937
School-level characteristics:		
School size: mean no. of pupils (SD)	205.1 (5.8)	185.4 (4.2)
Mean prevalence of malaria (range)	78.4% (54.0–94.1%)	80.3% (52.0–92.0%)
Individual-level characteristics:		
Mean age in years	9.4 (2.3)	9.5 (2.2)
Proportion Female	48.2% (460/954)	48.7% (456/937)
Nutritional status: height-for-age z-score	−0.85 (1.23)	−0.83 (1.48)
Nutritional status: BMI-for-age z-score	−0.68 (0.93)	−0.61 (0.97)
Father is literate*	51.9% (374/721)	53.9% (386/716)
Mother is literate*	20.1% (145/721)	18.5% (133/720)
Household has a moped*	76.4% (539/706)	83.9% (600/715)
Household has a telephone*	75.1% (527/702)	78.8% (564/716)
Child slept under a net night before survey*	41.4% (296/715)	41.9% (299/713)
Study endpoints at baseline: biomedical		
Prevalence of anaemia	62.6% (580/927)	63.2% (562/889)
Mean haemoglobin concentration, g/L	110.2 (13.1)	110.4 (13.5)
*Plasmodium* infection:†		
Prevalence of malaria parasites: all species	78.8% (685/869)	80.7% (725/899)
Parasite density per µL blood, all species in slide-positive children: geometric mean	693.8 (99.5)	492.0 (121.5)
Prevalence of gametocytes: all species	5.9% (51/869)	4.8% (43/899)

Data are mean (SD), or % (n/N), unless specified otherwise.

*Data based on child report by schoolchildren surveyed in classes 3–6; questions were not asked of younger children in classes 1 and 2.

†For three schools, many blood slides were inadvertently destroyed (two controls and one intervention), resulting in missing data for *Plasmodium* infection.

BMI, body mass index.

**Table 1b:** Characteristics of children in the cognitive cohort in control and intervention schools at baseline: November 2010 (classes 4 and 5 only)

	Control schools	Intervention schools
Number of schools surveyed	40	40
Number of children surveyed	600	597
School-level characteristics:		
School size: mean no. of pupils (SD)	167.3 (21.2)	158.5 (18.7)
Individual-level characteristics:		
Mean age in years (SD)	10.0 (1.2)	10.4 (1.4)
Proportion Female	47.0% (282/600)	44.2% (264/597)
Enrolled in class 5	27.5% (165/600)	37.3% (222/597)
Study endpoints at baseline: cognitive		
Code transmission: single-digit task, z-score	0.069 (1.00)	−0.069 (0.99)
Code transmission: double-digit task, z-score	0.035 (0.96)	−0.035 (1.04)
Visual search task, z-score	−0.049 (0.99)	0.049 (1.00)
Numeracy task, z-score	−0.013 (1.00)	0.013 (1.00)
Vocabulary task, z-score	0.029 (1.02)	−0.029 (0.97)
Writing task, z-score	0.037 (0.94)	−0.037 (1.05)

Data are mean (SD), or % (n/N), unless specified otherwise.

The trial profile for the cognitive cohort in 80 schools (classes 4 and 5 only) is shown in figure 1 (pink boxes); 99.5% of children enrolled into the cohort were examined in the post-intervention survey in February 2012. The number of children examined at each time point in the 38 schools included in the biomedical surveys (classes 1–6) is also shown in figure 1 (blue boxes).

### Intervention compliance

**Figure 2:**
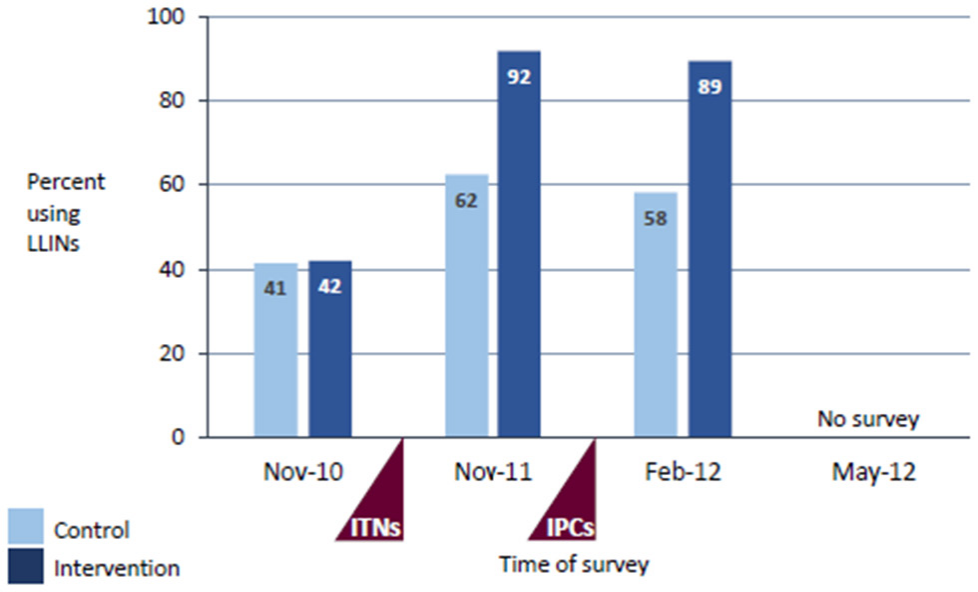
Percentage of schoolchildren who reported sleeping under a mosquito net the night before the survey at baseline, 12-month and 15-month follow-up.

Following the school-based and national universal community distribution of LLINs, reported net use among schoolchildren in August 2011 exceeded 80% in both intervention and control communities.^29^ The malaria prevention education increased both malaria knowledge and reported mosquito net use among school children, reaching a peak of 98% net use in intervention schools in August 2011. Differences in reported net use between the two groups increased with time, with 92% of children in intervention schools still using nets at the end of the transmission season in November 2011, compared with 62% in control schools (figure 2). A comparison of the recorded number of treatment doses administered with school enrolment showed coverage of the IPCs treatment administered by teachers was high, with an overall average of 94.6% of pupils receiving the treatment; coverage ranged from 89.5% to 100% between schools. There were a few adverse events reported by teachers, mainly stomach ache and vomiting immediately after treatment, with most symptoms being mild and self-limiting. No severe adverse effects related to the administration of AS/SP were reported.

### Impact on biomedical outcomes

**Figure 3:**
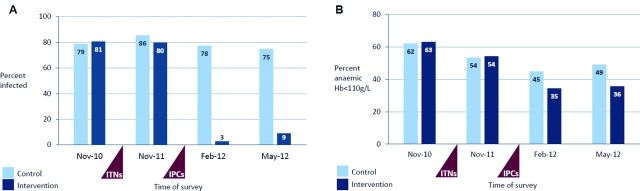
(A) Prevalence of malaria parasitaemia in control and intervention schools at baseline, 12-month, 15-month and 18-month follow-up. (B) Prevalence of anaemia in control and intervention schools at baseline, 12-month, 15-month and 18-month follow-up.

Changes in the prevalence of malaria parasitaemia and anaemia in control and intervention schools over time are shown in figure 3a and b. At follow-up in November 2011, after the first phase of the intervention (malaria prevention education and LLIN distribution), the prevalence of *Plasmodium* infection was slightly lower in children in intervention schools compared with controls. Since *Plasmodium* infection increased from 79% in November 2010 to 86% in November 2011 in the control arm, while remaining stable at around 80% across both time points in the intervention arm, the differential change since baseline between the two groups corresponds to a 44% reduction in the odds of *Plasmodium* infection in intervention schools compared with control schools (OR: 0.56; 95% CI 0.39 to 0.80, p=0.001, table 2). However, there was no evident difference in anaemia, haemoglobin concentration or gametocyte carriage between the two arms, after the first phase of the intervention.

**Table 2:** Effect of the intervention on health outcomes at 12-month and 15-month follow-up (classes 1–6)*

Trial endpoints	Summary statistics		Crude change from baseline		Effect estimate Intervention versus Control schools† OR/difference (95% CI); P value			
	Control schools	Intervention schools	Control schools	Intervention schools	Basic model‡		Fully adjusted model‡	
***Biomedical outcomes in November 2011:***after malaria prevention education in intervention schools, and LLIN distributions in both groups								
Anaemia	53.4% (502/940)	54.4% (506/931)	−9.1%	−8.8%	1.10 (0.76 to 1.58)	0.616	1.07 (0.72 to 1.58)	0.741
Mean Hb, g/L (SD)	113.5 (13.1)	114.4 (12.5)	+3.3	+4.0	0.45 (−1.03 to 1.93)	0.550	0.28 (−1.23 to 1.78)	0.719
*Plasmodium* infection								
Trophozoites, all species	85.6% (805/940)	79.9% (744/931)	+6.8%	−0.8%	0.56 (0.39 to 0.80)	0.001	0.59 (0.40 to 0.87)	0.007
Gametocytes, all species	9.2% (86/940)	8.1% (75/931)	+3.3%	+3.3%	1.04 (0.60 to 1.81)	0.887	0.98 (0.56 to 1.70)	0.929
***Biomedical outcomes in February 2012:*** after intermittent parasite clearance in intervention schools								
Anaemia	45.0% (418/930)	34.5% (321/931)	−17.6%	−28.7%	0.56 (0.40 to 0.78)	0.001	0.55 (0.39 to 0.80)	0.001
Mean Hb, g/L (SD)	117.2 (15.7)	120.5 (14.2)	+7.0	+10.1	2.80 (1.12 to 4.47)	0.001	2.66 (0.92 to 4.40)	0.003
*Plasmodium* infection								
Trophozoites, all species	77.5% (737/951)	2.8% (25/897)	−1.3%	−77.9%	0.005 (0.002 to 0.011)	<0.001	0.007 (0.003 to 0.014)	<0.001
Gametocytes, all species	5.7% (54/951)	0.1% (1/897)	−3.5%	−8.0%	0.02 (0.00 to 0.17)	<0.001	See footnote§	

Data are mean (SD), or % (n/N), unless specified otherwise.

*Includes data from children from all classes in the 38 schools included in the biomedical surveys; age range 5–18 years.

†Children in intervention schools received the educational intervention and an insecticide-treated net in April–May 2011, followed by intermittent parasite clearance in December 2011. Biomedical outcomes are compared with schoolchildren in control communities that had received nets as part of a government-run universal coverage campaign in May 2011.

‡All statistical analyses account for clustering within schools and repeated measures within individuals over time. Fully adjusted analyses control additionally for age, sex, height for age z-score and cluster level prevalence of malaria parasitaemia at baseline (proxy for geographical differences risk of infection).

§Not tested because of zero cell value due to incomplete covariate data for the gametocyte-positive child in intervention arm.

In contrast, at follow-up in February 2012 (8 weeks after the IPCs treatment), we found strong evidence for an impact of the intervention on asymptomatic *Plasmodium* infection, anaemia and haemoglobin concentration (all p≤0.001, table 2). The prevalence of asymptomatic parasitaemia fell to 3% in the intervention arm in February 2012 while remaining stable relative to baseline at 78% in the control arm, resulting in a 99% greater reduction in the odds of *Plasmodium* infection in intervention schools than control schools (OR: 0.005; 95% CI 0.002 to 0.011; p<0.001). After treatment, the prevalence of gametocytes was also lower in intervention schools compared with control schools that had not received treatment (0.1% vs 5.7%, respectively, p<0.001). Over the same period, the prevalence of anaemia fell in both arms of the study, but the reduction was greater in intervention schools: resulting in a 44% greater reduction in the odds of anaemia in intervention than control schools (OR: 0.56; 95% CI 0.40 to 0.78, p=0.001). The corresponding increase in haemoglobin levels was 2.8 g/L greater (95% CI 1.1 to 4.5) in intervention than control schools. Adjusted analyses that controlled for age, sex, height for age z-score and school-level prevalence of malaria parasitaemia at baseline (a proxy for geographical differences in the risk of infection) were broadly consistent with the unadjusted results reported above.

### Impact on cognitive outcomes

For cognitive outcomes, only the full intervention including the IPCs treatment was evaluated. Scores on both tests of sustained attention increased in all schools over the 15 months of the study, with the greatest improvements evident in the intervention arm (table 3). In analyses performed on the full cognitive sample, the mean z-score on the double-digit test increased by 0.23 points more in intervention than in comparison schools (95% CI 0.10 to 0.36), providing strong evidence for an impact of the intervention on sustained attention (p<0.001). The single-digit test score likewise increased slightly more in the intervention arm than the control arm, but the effect was small and non-significant (0.08 points; 95% CI −0.05 to 0.22). There was no evidence of any impact of the intervention on the other cognitive measures (visual search, numeracy, vocabulary or writing). Analyses adjusting for age, sex and school class were almost identical to the unadjusted results.

**Table 3:** Effect of the intervention on cognitive outcomes at 15-month follow-up (classes 4 and 5 only)*

Trial endpoints	Summary statistics		Crude change from baseline		Effect estimate: Intervention vs Control schools† Difference (95% CI); P value			
	Control schools	Intervention schools	Control schools	Intervention schools	Basic model‡		Fully adjusted model‡	
Cognitive outcomes in February 2012:								
Code transmission:								
Single-digit task, z-score	1.39 (1.00)	1.34 (1.00)	+1.32	+1.41	0.08 (−0.05 to 0.22)	0.223	0.08 (−0.06 to 0.21)	0.260
Double-digit task, z-score	0.37 (1.00)	0.53 (1.07)	+0.34	+0.57	0.23 (0.10 to 0.36)	<0.001	0.23 (0.10 to 0.36)	0.001
Visual search task, z-score§	0.74 (0.97)	0.81 (1.02)	+0.79	+0.76	−0.09 (−0.30 to 0.13)	n/s	−0.07 (−0.28 to 0.15)	n/s
Numeracy task, z-score§	0.66 (0.95)	0.66 (0.93)	+0.63	+0.69	0.00 (−0.15 to 0.15)	n/s	0.01 (−0.15 to 0.15)	n/s
Vocabulary task, z-score§	0.67 (1.41)	0.70 (1.35)	+0.68	+0.69	0.02 (−0.26 to 0.31)	n/s	0.00 (−0.28 to 0.30)	n/s
Writing task, z-score§	−0.20 (1.25)	−0.28 (1.43)	−0.24	−0.24	−0.07 (−0.47 to 0.32)	n/s	−0.07 (0.74 to −0.47)	n/s

Data are mean (SD) unless specified otherwise.

*Includes data from children in classes 4 and 5 recruited into the cognitive cohort in 80 schools, age range 6–16 years.

†Children in intervention schools received the educational intervention and an insecticide-treated net in April–May 2011, followed by intermittent parasite clearance in December 2011. Cognitive outcomes are compared with schoolchildren in control communities that had received nets as part of a government-run universal coverage campaign in May 2011.

‡All statistical analyses account for clustering within schools, repeated measures within individuals over time and practice effects. Fully adjusted analyses control for age, sex and school class.

§Analyses additionally control for different versions of the tasks administered to classes 4 and 5. 95% CI are from bias-corrected bootstrap analyses using 2000 bootstrap samples, and n/s indicates that the results are not statistically significant at p=0.05.

A supplementary analysis on the smaller subset of children for whom both biomedical and cognitive measures were available (shown in purple in figure 1), controlling additionally for parental literacy, socioeconomic status, height for age z-score and school-level prevalence of malaria parasitaemia at baseline, gave similar results to the analysis on the full sample (n=617, online supplementary table S2). In the fully adjusted analysis, the effect estimate for the single-digit test was close to that seen for the double-digit test; however, the sample size is small, and effects did not reach statistical significance (p=0.053 and p=0.057, respectively).

### Cross-sectional survey in May 2012

Children in control schools received intermittent parasite clearance after the endline survey in February 2012. To assess the longevity of the treatment effect on biomedical outcomes, an additional malariometric survey was undertaken at the end of the school year in May 2012 in a randomly selected subsample of 243 schoolchildren in the intervention arm, compared with 250 children in five schools that had not previously participated in the study. Children in these comparison schools had received no malaria programmes other than those in current national policy, including the community-based distribution of LLINs implemented in May 2011. The prevalence of *Plasmodium* infection and anaemia in May 2012 was comparable with the post-treatment prevalence measured in intervention and control schools in February 2012 (table 4), indicating that the effect of treatment on malaria and anaemia was sustained for up to 6 months post-treatment, effectively until the beginning of the next transmission season.

**Table 4:** Prolonged effect on biomedical outcomes at 6 months after parasite clearance*

	**Summary statistics**		
	**N (%)/mean (SD)**		
	**Non-intervention schools** **(did not participate in the trial)**	**Intervention schools** **(enrolled in trial 2010–2011)**	**P value**
Biomedical outcomes in May 2012:			
Anaemia	49.2% (123/250)	35.8% (87/243)	0.126
Mean Hb, g/L	114.46 (13.48)	118.84 (12.49)	0.109
*Plasmodium* infection:			
Trophozoites, all species	74.8% (187/250)	9.1% (22/243)	<0.0001
Gametocytes, all species	9.6% (24/250)	0.8% (2/243)	–

*Data from a cross-sectional survey at the end of the dry season of 493 children in random subsample of five intervention schools compared with children enrolled in five schools with no prior involvement in the study (50 children per school). Data are mean (SD), or % (n/N), unless specified otherwise.

## Discussion

This trial has shown that the combination of malaria prevention education, insecticide-treated nets and intermittent parasite clearance in schools can improve the health and cognitive performance of school children living in an area of highly seasonal transmission. Parasite clearance was associated with dramatic reductions in malaria parasitaemia and gametocyte carriage in intervention compared with control schools. This effect was sustained for 6 months until the beginning of the next transmission season. Malaria parasite clearance was also associated with a significant decrease in anaemia and increase in sustained attention. These findings from Mali are consistent with previous trial evidence^12 13^ and add to the limited but growing body of evidence on the impact of asymptomatic malaria infections on cognitive performance in schoolchildren.^6 12 14 18^

There were no marked differences in biomedical status at baseline or other socioeconomic characteristics between study groups, suggesting that treatment effects were not due to treatment bias. This was an open-label trial; nonetheless, we believe that the measures we took limited the scope for observation bias. Objective measures and standardised procedures were used during field surveys; the microscopists and staff marking cognitive tests were blind to the intervention status of schools; and data analysis was undertaken by a statistician with no prior involvement in the study and blind to intervention group. Since the advent of regular, countrywide deworming programmes in schools in Mali, the prevalence of helminth infection in this age group is generally low, and deworming was not carried out as part of this study. Stool examination in a subsample of 120 students in eight schools (four intervention and four control schools; 15 students per school) in January 2012 found <1% children to be parasite positive, confirming helminth infection to be uncommon in this study population though undernutrition may remain a major cause of anaemia. Nevertheless, the similarities in nutritional status between the two study groups at baseline would indicate that the effect on anaemia can most likely be attributed to the marked reduction in malaria parasitaemia. Finally, the findings are remarkably consistent with previous trials in Kenya, Mali, Senegal and Uganda, in which the protective efficacy of intermittent preventive treatment on anaemia in school-aged children has usually exceeded 40%.^12 13 18 19 22^ These data suggest that malaria control in schools could be beneficial across a wide range of transmission settings, from intense perennial transmission to highly seasonal transmission. Whether similar effects could be achieved in areas where the prevalence of *Plasmodium* infection and malaria-related anaemia in schoolchildren is lower remains to be seen.

Performance in cognitive tests can be expected to increase over time in both groups, due to the increasing age of the test subjects and repeated testing, which increases familiarity with the testing methodology (practice effects); nonetheless, the size of the improvement in sustained attention was greater among children in intervention schools. We did not see any differences between groups in any of the other tasks included in the cognitive battery. However, unlike the sustained attention task that only requires knowledge of the numbers 1–9, these other tasks are more dependent on prior learning (including reading, writing and knowledge of number sequences). The sustained attention task may therefore be less context dependent and more sensitive to changes in cognitive function. The ability to use the same test methodology with limited prior adaption also facilitates comparison with other recent studies.^12 18^ The effect size of 0.23 (95% CI 0.10 to 0.36) in improving test scores in sustained attention observed in this trial was lower than that seen in a previous trial in western Kenya, an area of intense perennial transmission with a lower EIR and lower prevalence of both *Plasmodium* infection and anaemia, where three rounds of parasite clearance were carried out per year.^12^ Whether this is due to differences in the number of treatment rounds, background level of malaria transmission, the aetiology of malaria or education between the two areas is unknown. Nonetheless, the finding that the intervention resulted in significant improvements in cognitive performance provides valuable corroborative evidence of the impact of malaria control on sustained attention in schoolchildren, and the potential for malaria control to support educational progress. In contrast to this mounting evidence on sustained attention,^12 18^ the effects of asymptomatic *Plasmodium* infection on other aspects of cognitive performance and education remain unknown. Whether these effects can be generalised to younger pupils at an earlier stage in their schooling is also unknown, and further research is needed.

In this study, we cannot fully separate the effects of each component of the intervention. Furthermore, the government campaign of universal LLIN coverage in May 2011 meant that children in both the intervention and control arms received a LLIN. Despite increases in malaria knowledge and reported net use in intervention schools following the school-based malaria education and LLIN distribution,^29^ the prevalence of *Plasmodium* infection remained high, and no impact was observed for haematological outcomes at the end of the transmission season. Although more children in the intervention arm, where the LLIN distribution was supported by focused malaria education activities in schools, reported using nets in November 2011, it is possible that they did not sleep under a net every night during the preceding months and/or were exposed to infective mosquitoes prior to going to bed. Increased and consistent net use would be expected to reduce the incidence of new infections during the malaria transmission season between May and October 2011; however, as demonstrated by the follow-up data from May 2012, asymptomatic infections can persist for several months, and the limited impact on malaria parasitaemia and anaemia may also have been due to long-term parasite carriage of infections acquired prior to the distribution of insecticide-treated nets. Thus, although substantial impacts were not seen until after the administration of antimalarial drugs for parasite clearance, we cannot rule out the role that net use might have played in the overall effect, through reducing the risk of new infections. It is possible that the effect of net use could be more marked in the subsequent transmission season, following the first annual round of treatment, but was not examined in this trial.

Qualitative studies undertaken after completion of the trial found the interventions to be well accepted by teachers, parents, school management committees and community leaders (Save the Children, unpublished data); the antimalarial treatment was generally well tolerated, with most symptoms being mild and self-limiting. The malaria prevention education component of the intervention was estimated to cost $2.13 per child, and the intermittent parasite clearance to cost an additional $2.72 per child treated.^30^ In this trial, a combination of SP/AS was used; however, the use of sulphadoxine-pyrimethamine in combination with amodiaquine as recommended by WHO for seasonal malaria chemoprevention in children under 5 years living in areas of highly seasonal transmission in the Sahel subregion^31^ would be preferable and would considerably reduce the cost of treatment. A drug combination with a longer half-life, such as dihydroartemisisin-piperaquine, can provide a longer period of protection against new infections^19^ but may be more costly and does not necessarily offer the same advantage in all epidemiological situations. Where IPCs is given at the end of the transmission season and infection risk is not ongoing, a drug combination with a shorter half-life might be sufficient.

Malaria transmission in the Sahel subregion, where this trial was carried out, is highly seasonal, with new infections concentrated in a few months each year. Schools are closed for most of the rainy season, which limits the role that schools can play in malaria control during the period of peak transmission. For example, as children are not in school in the months when the incidence of clinical attacks is highest, there is limited value in training teachers to diagnose and treat malaria. Our intervention package in schools instead focused on three complementary approaches that could be implemented in schools before and after the rainy season: (1) distribution of LLINs, accompanied by (2) malaria prevention education to promote net use and prompt treatment seeking for fever during the rainy season and school recess, followed by (3) parasite clearance at the end of the transmission season. A supplementary community-based approach to controlling malaria in school-aged children during the rainy season that might also be considered is the use of seasonal malaria chemoprevention, currently recommended for children under 5 years.^31^ Our study in schools predated this policy recommendation, and we did not evaluate this approach in older children. Nonetheless, our results are indicative of the potential value of extending the target age range of seasonal malaria chemoprevention to include older school-aged children.^32^

## Conclusion

Our findings add to the growing body of evidence on the impact of asymptomatic *Plasmodium* infection on cognitive performance in schoolchildren. This trial also provides further evidence that intermittent parasite clearance in schools is an efficacious control strategy to improve the health and cognitive performance of school-aged children. This approach is particularly suited to areas of seasonal transmission where a single annual treatment can be given at the end of the transmission season. Intermittent parasite clearance can be combined with the use of LLINs to reduce the risk of new infections between treatment rounds. In this area of seasonal transmission, malaria prevention education in schools encouraged continued use of nets for longer to ensure that children remain protected throughout the period of highest transmission risk. Malaria control initiatives in schools can also play a valuable role in supporting wider malaria control efforts. Malaria education activities in schools help to increase use of nets among schoolchildren and thereby contribute to higher levels of community coverage as well. The striking and sustained impact of a single round of intermittent parasite clearance on gametocyte carriage at the beginning of the next transmission season may have a further valuable effect in reducing the infectious reservoir that sustains transmission in areas of highly seasonal malaria.^33 34^ Improved malaria control in schools thus has the potential to generate both immediate and longer term benefits for schoolchildren, and for the communities in which they live.
