# Supplementary material for: Impact of a malaria intervention package in schools on Plasmodium infection, anaemia and cognitive function in schoolchildren in Mali: a pragmatic cluster-randomised trial
Source: BMJ Glob Health. 2017 Jun 28;2(2):e000182. doi: 10.1136/bmjgh-2016-000182 (PMC5656118; doi:10.1136/bmjgh-2016-000182)
Supplement: Supplementary Table 1 [file bmjgh-2016-000182supp001.pdf]

**Supplementary Table S1:** Characteristics of subset of children in cognitive cohort examined in biomedical surveys at baseline (classes 4 and 5 in 38 schools)

| | **Control schools** | **Intervention schools** |
|---|---|---|
| Number of schools surveyed | 19 | 19 |
| Number of children surveyed | 284 | 284 |
| ***School-level characteristics:*** | **Mean (SD)** | **Mean (SD)** |
| School size: mean no. of pupils | 205.1 (5.8) | 185.4 (4.2) |
| Mean prevalence of malaria (range) | 78.4% (54.0 to 94.1) | 80.2% (52.0 to 92.0) |
| ***Individual-level characteristics:*** | **Mean (SD)** | **Mean (SD)** |
| Mean age in years | 10.1 (1.1) | 10.4 (1.3) |
| Female (%) | 46.1% (131 / 284) | 45.4% (129 / 284) |
| Enrolled in class 5 (%) | 21.1% ( 60 / 284) | 26.4% ( 75 / 284) |
| Nutritional status: Height-for-age z-score | -0.82 (1.20) | -1.06 (1.49) |
| Nutritional status: BMI-for-age z-score | -0.71 (0.81) | -0.84 (0.82) |
| Father is literate[1] (%) | 73.4% (207 / 282) | 70.3% (199 / 283) |
| Mother is literate[1] (%) | 32.3% ( 91 / 282) | 23.2% ( 66 / 284) |
| Household has a moped[1] (%) | 75.4% (199 / 264) | 80.6% (224 / 278) |
| Household has a telephone[1] (%) | 75.0% (198 / 264) | 82.4% (230 / 279) |
| Child slept under a net night before survey[1] (%) | 42.1% (111 / 264) | 43.0% (120 / 279) |
| ***Study endpoints at baseline: Biomedical*** | **Mean (SD)** | **Mean (SD)** |
| Prevalence of anaemia: Hb <110 g/L (%) | 57.2% (159 / 278) | 57.4% (160 / 279) |
| Mean haemoglobin concentration, g/L | 111.1 (12.9) | 112.6 (12.7) |
| *Plasmodium* infection: | | |
| Prevalence of malaria parasites: all species (%) | 78.7% (203 / 258) | 79.1% (216 / 273) |
| Parasite density per µL blood, all species in slide-positive children: geometric mean | 654.4 (4.6) | 449.2 (4.5) |
| Prevalence of gametocytes: all species (%) | 3.5% ( 9 / 258) | 4.4% (12 / 273) |
| ***Study endpoints at baseline: Cognitive*** | **Mean (SD)** | **Mean (SD)** |
| Code transmission: single digit task, z-score | 0.075 (1.05) | -0.12 (0.94) |
| Code transmission: double digit task, z-score | 0.024 (0.85) | -0.044 (1.06) |
| Visual search task, z-score | -0.019 (0.92) | 0.041 (1.03) |
| Numeracy task, z score | -0.013 (1.00) | 0.099 (1.00) |
| Vocabulary task, z-score | 0.016 (1.05) | -0.010 (0.96) |
| Writing task, z-score | 0.023 (0.92) | -0.015 (1.06) |

Data are mean (SD), or % (n/N), unless specified otherwise.

[1] Data based on child report

**Supplementary Table S2:** Effect of the intervention on biomedical and cognitive outcomes at 15 months follow-up: Results in subset of children in cognitive cohort with data on biomedical outcomes and household environment (classes 4 and 5 in 38 schools)

| Trial endpoints | Summary Statistics | | Crude Change from Baseline | | Effect estimate: Intervention vs Control schools. Odds ratio / Difference (95% CI); *p*-value | | | |
|---|---|---|---|---|---|---|---|---|
| | Control schools | Intervention schools | Control schools | Intervention schools | Basic Model[1] | | Fully Adjusted Model[1] | |
| **Biomedical outcomes[2] in February 2012** | | | | | | | | |
| Anaemia: Hb<110 g/L | 38.6% (103 / 267) | 36.7% (101 / 275) | -18.6 | -20.7 | 0.91 (0.51 to 1.60) | 0.733 | 0.77 (0.42 to 1.43) | 0.413 |
| Mean Hb, g/L | 119.4 (16.3) | 120.7 (13.5) | +8.3 | +8.1 | -0.08 (-2.79 to 2.63) | 0.955 | -0.40 (-3.26 to 2.46) | 0.783 |
| *Plasmodium* infection: | | | | | | | | |
| Trophozoites, all species | 73.0% (200 / 274) | 4.0% (11 / 276) | -5.7 | -75.1 | 0.012 (0.00 to 0.03) | <0.001 | 0.012 (0.004 to 0.033) | <0.001 |
| Gametocytes, all species | 4.4% ( 12 / 274) | 0.4% ( 1 /276) | +0.9 | -4.0 | 0.06 (0.01 to 0.58) | 0.015 | - | - |
| **Cognitive outcomes[3] in February 2012** | | | | | | | | |
| *Code transmission*: | | | | | | | | |
| Single digit task, z-score | 1.43 (0.96) | 1.32 (0.93) | +1.36 | +1.44 | 0.03 (-0.16 to 0.22) | 0.774 | 0.21 (0.00 to 0.41) | 0.053 |
| Double digit task, z-score | 0.42 (0.97) | 0.58 (1.01) | +0.40 | +0.62 | 0.22 (0.05 to 0.39) | 0.014 | 0.19 (-0.01 to 0.39) | 0.057 |
| Visual search task[4], z-score | 0.75 (1.03) | 0.74 (1.00) | +0.77 | +0.70 | -0.06 (-0.39 to 0.31) | n/s | 0.06 (-0.27 to 0.43) | n/s |
| Numeracy task[4], z-score | 0.74 (0.93) | 0.66 (0.93) | +0.75 | +0.56 | -0.11 (-0.32 to 0.12) | n/s | -0.09 (-0.36 to 0.16) | n/s |
| Vocabulary task[4], z-score | 0.66 (1.31) | 0.73 (1.26) | +0.64 | +0.74 | 0.08 (-0.38 to 0.52) | n/s | 0.06 (-0.43 to 0.59) | n/s |
| Writing task[4], z-score | -0.23 (1.33) | -0.38 (1.42) | -0.25 | -0.37 | -0.09 (-0.77 to 0.56) | n/s | -0.19 (-0.87 to 0.50) | n/s |

Data are mean (SD), or % (n/N), unless specified otherwise.

1 All statistical analyses account for clustering within schools and repeated measures within individuals over time.

2 Fully adjusted analyses of biomedical outcomes control for age, sex, height for age z-score, ownership of household assets (proxy for socioeconomic status), and cluster-level prevalence of malaria parasitaemia at baseline (proxy for geographical differences risk of infection).

3 All analyses of cognitive outcomes control for practice effects. Fully adjusted analyses control additionally for age, sex, school class, height for age z-score, parental literacy, ownership of household assets, and cluster- level prevalence of malaria parasitaemia at baseline.

4 Analyses of these tasks additionally control for different versions of the tasks administered to classes 4 and 5. 95% CI are from bias-corrected bootstrap analyses using 2,000 bootstrap samples and n/s indicates that the results are not statistically significant at p=0.05.
